# Rolling Out Behavioral Recovery Outreach (BRO) Teams: Perspectives From an Inaugural Partner Site

**DOI:** 10.1093/geroni/igab046.175

**Published:** 2021-12-17

**Authors:** Trisha Gaudig

**Affiliations:** Sioux Falls VA, Sioux Falls, South Dakota, United States

## Abstract

The Sioux Falls VA Community Living Center (CLC) is a partner site for the Behavioral Recovery Outreach (BRO) Team dissemination. This CLC is home to over 55 Veterans requiring a variety of specialty needs such as dementia care, short-term rehabilitation, respite, hospice, and/or psychosocial needs. Many of the Veterans followed by the BRO Team on the CLC experienced frequent rehospitalizations and difficult placement in the community due to behavioral concerns. Local leadership encouraged participation in the BRO Team dissemination due to the growing need in this VA system to open access to dementia and mental health care, successfully discharge Veterans to appropriate community settings, and reduce unnecessary rehospitalizations. This presentation will discuss BRO Team development, including several factors facilitating successful BRO Team implementation (e.g., leadership support, community outreach approaches, staff partner buy-in), and identify barriers impacting successful implementation with a case example to illustrate strategies to overcome such barriers.

